# Identification of Human Herpesvirus 8 Sequences in Conjunctiva Intraepithelial Neoplasia and Squamous Cell Carcinoma of Ugandan Patients

**DOI:** 10.1155/2015/801353

**Published:** 2015-10-05

**Authors:** Noemy Starita, Clorinda Annunziata, Keith M. Waddell, Luigi Buonaguro, Franco M. Buonaguro, Maria Lina Tornesello

**Affiliations:** ^1^Molecular Biology and Viral Oncology, Istituto Nazionale Tumori “Fondazione G Pascale”, IRCCS, 80131 Naples, Italy; ^2^Departments of Ophthalmology and Paediatrics, Mbarara University of Science and Technology, P.O. Box 1410, Mbarara, Uganda

## Abstract

The incidence of squamous cell carcinoma of the conjunctiva is particularly high in sub-Saharan Africa with temporal trends similar to those of Kaposi sarcoma (KS). Human herpesvirus type 8 (HHV8), has not yet been investigated in conjunctiva tumors. In this study biopsies and PBMCs of conjunctiva neoplasia patients along with nonneoplastic conjunctiva tissues have been analyzed for HHV8 sequences by PCR targeting ORF26. All amplimers were subjected to nucleotide sequencing followed by phylogenetic analysis. HHV8 DNA has been identified in 12 out of 48 (25%) HIV-positive, and in 2 out of 24 (8.3%) HIV-negative conjunctiva neoplastic tissues and in 4 out of 33 (12.1%) PBMC samples from conjunctiva neoplasia diseased patients as well as in 4 out of 60 (6.7%) nontumor conjunctiva tissues. The viral load ranged from 1 to 400 copies/10^5^ cells. Phylogenetic analysis showed that the majority of HHV8 ORF26 amplimers clustered with subtypes R (*n* = 11) and B2 (*n* = 6). This variant distribution is in agreement with that of HHV8 variants previously identified in Ugandan KS cases. The presence of HHV8 in conjunctiva tumors from HIV-positive patients warrants further studies to test whether HHV8 products released by infected cells may have paracrine effects on the growth of conjunctiva lesions.

## 1. Introduction

The incidence of squamous cell carcinoma of the conjunctiva (CSCC) has shown a dramatic increase in the sub-Saharan African populations during the HIV/AIDS era [1–5]. In Uganda the incidence has increased more than tenfold between 1960–1971 and 1995–1997 and has remained high during the period 1991–2010 [6]. Similarly, a 10-fold increase in the incidence of conjunctival carcinoma has been reported in Harare, Zimbabwe, during the period 1991–2004 [3]. This finding supported the hypothesis that HIV-related immune suppression could facilitate the oncogenic process of other oncogenic agents infecting the conjunctival mucosa. Several viruses have been searched in HIV-positive and HIV-negative conjunctival neoplasia, including cutaneous and mucosal human papillomaviruses [7–9], but the etiologic mechanism of such tumor remains still unclear.

Human herpesvirus type 8 (HHV8) is the causal agent of all clinical forms of Kaposi sarcoma, of two B-cell tumors, namely, primary effusion lymphoma and multicentric Castleman disease, and the recently described HHV8 inflammatory cytokine syndrome [10–14]. Kaposi sarcoma is a vascular lesion which frequently develops in mucocutaneous sites including the ocular surface [15–17]. Indeed, Kaposi sarcoma of the conjunctiva and ocular adnexa were observed in approximately 5% of HIV/AIDS patients before HAART [18].

The HHV8 encodes several homologues of human proteins, such as viral G protein-coupled receptor (vGPCR), viral interferon regulatory factors 1–4 (vIRF 1–4), viral interleukin 6 (vIL-6), viral Fas-associated death domain-like IL-1-converting enzyme inhibitory protein (vFLIP), and vBCL2 that are able to promote cell survival, immune evasion, angiogenesis, and inflammation [19]. Moreover, HHV8 vGPCR induces secretion of vascular endothelial growth factor (VEGF), IL-6, and platelet derived growth factor (PDGF) which, together with the vIL-6 and vFLIP, deregulate via autocrine and paracrine mechanisms the proliferation and apoptosis of uninfected cells surrounding those harboring replicating virus [20, 21]. Moreover, in the HHV8 inflammatory cytokine syndrome the symptoms are associated with excess lytic activation of the virus, elevated levels of HHV8 vIL-6, IL-6, and viral loads [22].

The HHV8 has been shown to infect a variety of cells including endothelial, epithelial, and B cells as well as monocytes and CD34+ hematopoietic progenitor stem cells [23, 24]. The viral DNA has been found in normal skin, plasma, and PBMCs of a significant fraction of Kaposi sarcoma patients [25]. In Uganda, where Kaposi sarcoma is endemic, HHV8 in plasma was detected in 8.7% of the general population [26]. Among HIV-positive patients with no diagnosis of Kaposi sarcoma, HHV8 DNA has been identified in PBMCs in 13% of patients in association with lower CD4+ cell counts and higher plasma HIV RNA [27].

DNA sequences of HHV8 have been also detected in non-Kaposi skin lesions of transplant recipients, in pemphigus vulgaris and mycosis fungoides lesions, in angiosarcomas, and in angiolymphoid hyperplasia [28]. No study systematically searched for HHV8 DNA in conjunctival neoplastic lesions.

This study aimed to analyze the prevalence of HHV8 DNA in conjunctival neoplasia biopsies at different stages of malignancy, including conjunctival intraepithelial lesions grades 1 to 3 (CIN1, -2, or -3), in invasive conjunctival squamous cell carcinoma (CSCC) and in PBMCs from conjunctiva neoplasia HIV-positive and HIV-negative patients as well as in conjunctival tissues from healthy control subjects.

## 2. Materials and Methods

### 2.1. Patients and Specimens

Conjunctival biopsies from 72 patients with conjunctival neoplasia from 60 conjunctiva subjects with nonneoplastic were obtained at seven countrywide eye clinics in Southern Uganda from all subjects who gave informed consent to participate in the study. Peripheral blood mononuclear cells (PBMCs) obtained from 33 conjunctiva neoplasia patients, whose surgical biopsies were not available, were also included in the study. The study protocol was approved by the local ethical review board. All cases and controls were previously characterized in terms of histology, DNA quality, HIV serology, and cutaneous and mucosal HPV DNA positivity [7, 29]. DNA extraction was performed with similar procedures for both types of samples (frozen biopsies and PBMCs). Briefly samples were digested with proteinase K (150 *μ*g/mL at 60°C for 30 min) in lysis buffer (10 mM Tris-HCl pH 7.6, 5 mM EDTA, 150 mM NaCl, and 1% SDS), followed by DNA purification with phenol and phenol-chloroform-isoamyl alcohol (25 : 24 : 1) extraction and ethanol precipitation in 0.3 M sodium acetate (pH 4.6).

### 2.2. PCR Amplification of HHV8 ORF26

The HHV8 ORF26 was amplified by nested PCR using oligoprimers and reaction conditions previously described [31, 32]. In particular, rightward ORF26 has been amplified with outer oligonucleotides LGH2574L (5′-CAGAAACAGGGCTAGGTAC-3′) and LGH2575R (5′-GTGCTTGACGATCTGTCC-3′) and with inner oligonucleotides SJF (5′-CTATCTTCAGAGTCTCAG-3′) and SJR (5′-TAGGTACACACAATTTTG-3′); leftward ORF26 has been amplified with outer oligonucleotides LGH1701R (5′-GGATCCCTCTGACAACC-3′) and SJ2R (5′-GCCAAGATTAAATATAGAACTGAG-3′) and inner oligonucleotides LGH1701R and SJ1R (5′-AATATAGAACTGAGACTCTGAAG-3′) (Table 1). PCR amplification reactions were performed in 50 *μ*L reaction mixture containing 100 to 300 ng of target DNA, 5 pmol of each primer, 2.5 mM MgCl_2_, 50 *μ*M of each dNTP, and 5 *μ*L Hot Master buffer and 2.5 U of Hot Master Taq DNA Polymerase (5 Prime GmbH, Hamburg, Germany). DNA was amplified in a Perkin-Elmer GeneAmp PCR System 9700 thermal cycler with the following steps: an initial 2 min denaturation at 94°C, followed by 45 amplification cycles of 55°C for 45 sec, 68°C for 1 min, 94°C for 15 sec, and a 5 min final elongation at 68°C. A reaction mixture containing genomic DNA, extracted from NIH 3T3 murine cell line, was used as negative control and was included in every set of 5 clinical specimens. All HHV8 amplimers were subjected to bidirectional direct sequencing analysis.

### 2.3. HHV8 Real Time PCR

A SYBR Green real time PCR method was used to determine HHV8 viral load in all DNA samples. Specially, oligoprimers ORF26LR1F1 (5′-GCAGTATCTATCCAAGTG-3′) and ORF26LR2R2 (5′-ACAGATCGTCAAGCA-3′) producing a 434 bp product were designed with Beacon Designer software (Premier Biosoft) and used for real time PCR (Table 1). HHV8 viral load quantization was performed in the Bio-Rad CFX96 real time PCR Detection System using 300 ng of template DNA, 12.5 *μ*L of iQ SYBR Green supermix (Bio Rad), and 5 pmol each of forward and reverse primers in a final volume of 25 *μ*L. Thermal cycling consisted of a denaturation step at 95°C for 3 min, followed by 50 cycles of 55°C annealing for 30 s, 72°C extension for 30 s, and 95°C denaturation for 30 s. A standard curve was constructed using serial dilutions (1.0 to 10^7^ copies of BCBL1 cell line, containing 70 copies per cell of HHV8 DNA). Three replicates were performed for each sample and real time PCR data were analyzed using BioRad CFX manager software. The averaged copy numbers in samples were calculated according to the standard curve and were represented as copies of viral DNA per 10^5^ cells. The amount of human genomic DNA in each sample was also determined by real time PCR targeting human *β*-globin gene (GH20, 5′-GAAGAGCCAAGGACAGGTAC-3′, and PC04, 5′-CAACTTCATCCACGTTCACC-3′) and the quantification of human *β*-globin gene was used to normalize the target DNA.

### 2.4. Nucleotide Sequencing and Phylogenetic Analysis

Aliquots of HHV8 PCR amplified products were subjected to bidirectional direct sequencing analysis by Eurofins Genomics (Milan, Italy) using the fluorescent dye terminator technology and ABI 3730 DNA sequencers (Applied BioSystems, Foster City, CA). This Sanger based technique is capable of detecting mixtures of viral variants when each variant represents >15% of the viral population. Nucleotide sequences were edited with Chromas Lite 2.01 (http://www.technelysium.com.au/chromas.html) and converted to FASTA format. Multiple sequence alignments of HHV8 sequences from the present study and reference strains reported in the GenBank were performed with clustal W tool of MegAlign program of the Lasergene software (DNASTAR Inc., V7.0.0). Reference sequences for each HHV8 ORF26 subtype were DQ984689.1 (BCBLR, A/C), DQ984768.1 (HKS15, R), DQ984785.1 (431K, B1), DQ984789.1 (021K, B2), and DQ984759.1 (HKS21, J).

### 2.5. Statistical Analysis

Statistical analysis was performed with Epi Info 6 Statistical Analysis System software (Version 6.04b, 1997, Centers for Disease Control and Prevention, USA). Unpaired *t*-test was used for comparisons of continuous variables (i.e., age); Mantel-Haenszel corrected *χ* ^2^ test and, where appropriate, two-sided Fisher's exact test were used for comparison of categorical data. Differences were considered to be statistically significant when *P* values were less than 0.05.

## 3. Results

Overall, HHV8 DNA has been detected in 14 out of 72 (19.4%) conjunctiva neoplastic tissues and in 4 out of 60 (6.7%) control tissues (Table 2). The prevalence of HHV8 DNA was found to be significantly different between cases and controls (*P* = 0.034). Following stratification of patients by HIV status the HHV8 DNA was found in 12 out of 48 (25%) HIV-positive and in 2 out of 24 (8.3%) HIV-negative conjunctiva neoplastic tissues (Table 3). The difference in HHV8 prevalence between these two groups was of borderline significance (Fisher's exact test two-tailed *P* = 0.120), due to the limited sample size of controls with known HIV serostatus. HHV8 DNA has been identified in 4 out of 33 (12.1%) PBMC samples of conjunctiva neoplasia diseased patients, for which conjunctiva biopsies were not available. The histology of the 72 conjunctival neoplasia patients identified 24 (33.3%) lesions as invasive conjunctiva squamous carcinoma, 17 (23.6%) as conjunctiva intraepithelial neoplasia grade 3 (CIN3), 16 (22.2%) as CIN2, and 15 (20.8%) as CIN1. The frequency of HHV8 infection among cases, grouped by histological types, was 20.8% (5/24) in the CSCC, 23.5% (4/17) in the CIN3, 25% (4/16) in CIN2, and 6.7% (1/15) in CIN1. Therefore, HHV8 detection frequency seems not associated with more advanced disease stages.

To quantify HHV8 viral load in conjunctiva DNA samples, serial dilution of DNA extracted from BCBL-1 cell line (range, 1 × 10^0^ to 1 × 10^6^ cells) in the background of human DNA was amplified with HHV8 and human *β*-globin oligonucleotides by real time PCR. The estimated HHV8 copy number was 70 per BCBL-1 cell, which is in agreement with the value reported in the literature [33]. Of the 72 conjunctiva neoplasia DNA samples tested by real time PCR, 18 (25%) were positive for HHV8 DNA with viral loads ranging from 1 to 400 copies/10^5^ cells in different samples.

Amplimers obtained by nested PCR from 17 DNA samples were sequenced across the rightward and leftward ORF26 locus. All nucleotide differences between samples are described in Table 4. HHV8 ORF26 sequences mainly belong to B2 (10 out of 17, 58.8%), R (5 out of 17, 29.4%), B1 (1 out of 17, 5.9%), and J (1 out of 17, 5.9%) subtypes, following the nomenclature proposed by Zong et al. (2007) [30]. No multiple infections with different HHV8 variants were identified by nucleotide sequencing analysis.

## 4. Discussion

HHV8 has been clearly associated with proliferative disorders such as Kaposi sarcoma, primary effusion lymphoma, and multicentric Castleman's disease [10]. Several viral genes, such as v-GPCR, vIRF 1–4, vFLIP, and vIL-6, mainly expressed during the lytic phase of viral replication have been recognized as transforming factors acting through autocrine and paracrine mechanisms [21, 34]. In fact, it has been shown that in Kaposi sarcoma tumors only few HHV8 infected cells undergo lytic reactivation and express a large number of viral proteins, which in turn contribute to angiogenesis in Kaposi tumors promoting the secretion of cellular or viral factors in a paracrine manner [21]. These paracrine proangiogenic properties raise the question whether HHV8 might be implicated in the pathogenesis of vascular proliferative lesions other than Kaposi sarcoma.

In this study HHV8 sequences have been identified in 25% and 8% of HIV-positive and HIV-negative conjunctival neoplasia samples, respectively, suggesting a major effect of HIV-related immune suppression in HHV8 replication. The phylogenetic analysis of the conserved ORF26 amplimers indicated that subtypes R and B2 were the most common variants in conjunctiva samples, in agreement with previous results on HHV8 variant distribution in Ugandan Kaposi sarcoma cases [32]. Furthermore, the similar frequency rate of HHV8 positivity in invasive tumors compared to CIN2 and CIN3 lesions suggests that the virus might be involved in early phases of tumor development but is unlikely involved in tumor progression.

HHV8 loads have been evaluated in conjunctiva neoplasia samples and found to be in the range of 1 to 400 copies/10^5^ cells. These values are comparable to those observed in PBMCs of HIV-related Kaposi sarcoma patients and are probably correlated to the immune status of subjects [35]. The results obtained so far are not sufficient to differentiate whether HHV8 has a direct role in the development of conjunctival carcinoma or is a bystander vehiculated by infected PBMCs in the high vascularized conjunctival lesions. However, in both cases it is possible to postulate a paracrine effect of the viral products enhancing angiogenesis and tumorigenesis in conjunctival mucosa.

The presence of HHV8 DNA has been investigated in several other disorders with controversial results. Nishimoto et al. identified HHV8 DNA sequences in a variable fraction of skin lesions such as Bowen's disease, actinic keratoses, leukoplakia, Paget's disease, melanoma, neurofibroma, and chronic dermatitis [36]. However, several other studies failed to confirm such association [37, 38].

McDonagh et al. described the presence of HHV8 DNA sequences in vascular proliferations including angiosarcomas (29%) and haemangiomas (5%) [39]. However, Lebbe et al. reported no association between HHV8 and non-Kaposi sarcoma vascular lesions in their patients [40].

More recently, few studies reported the detection of low levels of HHV8 DNA in lymphoproliferative diseases such as large-plaque parapsoriasis and mycosis fungoides [41, 42], but not confirmed in other studies [43–45]. Kreuter et al. hypothesized that this association may depend on high HHV8 seroprevalence in some geographic regions or HHV8 reactivation in immune compromised patients [42].

Moreover, Nalwoga et al. have recently shown that in Uganda, where malaria is highly endemic, the positivity for malaria antibodies is strongly associated with HHV8 seropositivity suggesting that malaria exposure may facilitate HHV8 reactivation, viral transmission, and related diseases [46].

Seroprevalence of HHV8 in Uganda is very high, ranging from 36% to 60% in the general population [47–49]. More recently high prevalence of HHV8 DNA has been detected in the plasma of HHV8 seropositive Ugandan subjects enrolled in a HIV/AIDS survey [26]. In their study, Shebl et al. found plasma viral DNA in 14% of HHV8 seropositive and 2% of HHV8 seronegative subjects suggesting that replicating virus is very common in the blood of Ugandan subjects, where HHV8 infection and Kaposi sarcoma are endemic.

This study has several limitations including the small sample size of controls with known HIV serostatus and the lack of material to evaluate HHV8 mRNA levels in conjunctiva samples. However this is the first study designed to systematically detect HHV8 DNA sequences in conjunctiva neoplasia at different grade of malignancy and the obtained results warrant further epidemiological and molecular studies.

## 5. Conclusions

In conclusion, HHV8 DNA sequences are detected in a significant fraction of HIV-positive conjunctival neoplasia cases from Uganda. The results obtained so far are not sufficient to determine whether HHV8 is a bystander vehiculated by infected PBMCs in the high vascularized conjunctival lesions or is able to infect cells populating the conjunctiva. Further studies are needed to determine if the expression and secretion of viral and human inflammatory factors, such as vIL6 and the human homologue, contribute to angiogenesis and tumorigenesis of conjunctival neoplasia in a paracrine fashion.

## Figures and Tables

**Table 1:** PCR primer sequences used to amplify HHV8 ORF26 regions by standard PCR and real time PCR.

Method	Locus	Primer name	Sequences (5′-3′)	Nucleotide position	Size	Reference
PCR outer	ORF26-3′	LGH2575-R	GTGCTTGACGATCTGTCC	47,638–47,655	620 bp	[30]
		LGH2574-L	CAGAAACAGGGCTAGGTAC	48,239–48,257		

PCR inner	ORF26-3′	SJ-F	CTATCTTCAGAGTCTCAG	47,844–47,861	402 bp	[31]
		SJ-R	TAGGTACACACAATTTTG	48,228–48,245		

PCR outer	ORF26-5′	LGH1701R	GGATCCCTCTGACAACC	47,292–47,309		
		SJ-R2	GCCAAGATTAAATATAGAACTGAG	47,857–47,880	589 bp	[31]

PCR inner	ORF26-5′	LGH1701R	GGATCCCTCTGACAACC	47,292–47,309		
		SJ-R1	AATATAGAACTGAGACTCTGAAG	47,848–47,870	579 bp	[31]

Real time PCR	ORF26-3′	ORF26LR1F1	GCAGTATCTATCCAAGTG	47,220–47,237		
		ORF26LR2R2	ACAGATCGTCAAGCA	47,639–47,653	434 bp	This study

Nucleotide positions are given on the GenBank sequence number NC_009333.

**Table 2:** Distribution of known variables between conjunctival neoplasia cases and controls.

	Conjunctiva neoplastic tissues	Conjunctiva control tissue^*∗*^	*P* value
	*N* = 72 (%)	*N* = 60 (%)	
Sex			0.724
M	31 (43.1)	24 (40.0)	
F	41 (56.9)	36 (60.0)	

Age			0.584
≤30 years	31 (43.1)	23 (38.3)	
>30 years	41 (56.9)	37 (61.7)	

HHV8 PCR			0.034
Positive	14 (19.4)	4 (6.7)	
Negative	58 (80.5)	56 (93.3)	

HIV serology^†^			0.004
Positive	48 (66.7)	15 (38.5)	
Negative	24 (33.3)	24 (61.5)	

^*∗*^Two pingueculae, 1 pterygium, and 1 papilloma of the conjunctiva were included in the control group.

^†^21 control subjects with undetermined HIV serology were not included.

**Table 3:** Distribution of HHV8 in HIV-positive and HIV-negative conjunctival neoplasia samples and controls.

HIV status	Conjunctiva neoplastic tissues	Conjunctiva control tissue^*∗*^	*P* value
	*N* (%)	*N* ^†^ (%)	
HIV positive			0.347^‡^
HHV8-Pos	12 (25.0)	2 (13.3)	
HHV8-Neg	36 (75.0)	13 (86.7)	

HIV negative			1.000^‡^
HHV8-Pos	2 (8.3)	1 (4.2)	
HHV8-Neg	22 (91.7)	23 (95.8)	

^*∗*^Two pingueculae, 1 pterygium, and 1 papilloma of the conjunctiva were included in the control group.

^†^21 control subjects with undetermined HIV serology were not included.

^‡^Fisher's exact test, two-tailed.

**Table 4:** Nucleotide changes identifying distinct subgroups of HHV8 genomes within the ORF26 gene locus. Dashes indicate identities to the prototype. Absence of genetic variations relative to the references is marked with dashes, whereas presence of variant nucleotides is indicated by the nucleotide corresponding letter. An empty space indicates sequence not available. ∧ indicates nucleotide insertion; *δ* indicates nucleotide deletion. The numbering system conforms to that used by Tornesello et al. [32].

Sample name			1	1	1	1	1	1	1	1	1	1	1	1	1	1	1	1	1	1	1	1	1	1	1	ORF26 class
	7	9	0	0	0	1	1	1	1	1	1	2	4	5	5	5	5	5	7	7	7	7	8	8	8	
	3	8	3	5	8	2	3	3	4	5	7	0	9	4	6	6	6	9	1	2	5	9	0	1	2	
	3	1	2	5	6	2	2	9	1	8	7	6	0	5	2	8	9	4	7	3	4	5	2	6	5	

BCBLR	G	T	C	G	C	G	A	A	A	C	C	G	G	C	∧	G	G	G	C	C	C	G	G	T	G	A/C

HKS15		C	—	—	T	—	—	C	—	—	—	—	T	—	0	*δ*	*δ*	T	T	—	—	—	—	—	—	R
CIN3.110	—	C	—	—	T	—	—	C	—	—	—	—	T	—												R
CIN3.179	—	C	—	—	T	—	—	C	—	—	—	—	T	—												R
CIN2.136	—	C	—	—	T	—	—	C	—	—	—	—	T	—												R
CIN2.169	—	C	—	—	T	—	—	C	—	—	—	—	T	—	0	*δ*	*δ*	T	T	—	—	—	—	—	—	R
CIN1.173	—	C	—	—	T	—	—	C	—	—	—	—	T	—	0	*δ*	*δ*	T	T	—	—	—	—	—	—	R

431K		C	—	—	—	—	G	C	—	—	—	—	—	—	0	T	A	—	—	T	—	C	—	C	A	B1
SCC.193	—	C	—	—	—	—	G	C	—	—	—	—	—	—	0	T	A	—	—	T	—	C	—	C	A	B1

021K		C	A	—	—	—	G	C	—	—	—	—	—	—	0	T	A	—	—	T	—	C	—	C	A	B2
SCC.108	—	C	A	—	—	—	G	C	—	—	—	—	—	—	0	T	A	—	—	T	—	C	—	C	A	B2
SCC.144	—	C	A	—	—	—	G	C	—	—	—	—	—	—												B2
SCC.218	—	C	A	—	—	—	G	C	—	—	—	—	—	—												B2
CIN3.99	—	C	A	—	—	—	G	C	—	—	—	—	—	—												B2
CIN3.167	—	C	A	—	—	—	G	C	—	—	—	—	—	—												B2
CIN2.138	—	C	A	—	—	—	G	C	—	—	—	—	—	—												B2
CIN2.143	—	C	A	—	—	—	G	C	—	—	—	—	—	—	0	T	A	—	—	T	—	C	—	C	A	B2
CTR.142	—	C	A	—	—	—	G	C	—	—	—	—	—	—												B2
CTR.143	—	C	A	—	—	—	G	C	—	—	—	—	—	—												B2
CTR.149	—	C	A	—	—	—	G	C	—	—	—	—	—	—												B2

HKS21		C	A	T	—	—	G	C	—	—		—	—	—	0	—	—	—	—	—	—	—	—	—	—	J
SCC.142	—	C	A	T	—	—	G	C	—	—	—	—	—	—												J

## Abbreviations

HHV8: Human herpesvirus type 8
CIN: Conjunctival intraepithelial neoplasia
CSCC: Conjunctival squamous cell carcinoma
KS: Kaposi sarcoma
HIV: Human immunodeficiency virus.
